# A new species of *Laccobius* Erichson, 1837 (Hydrophilidae, Coleoptera) from the Chinese Himalaya, with comments on taxonomic status of subgenera *Glyptolaccobius* Gentili, 1989 and *Cyclolaccobius* Gentili, 1991 and additional faunistic records from China

**DOI:** 10.3897/zookeys.889.34690

**Published:** 2019-11-14

**Authors:** Fenglong Jia, Jia-Hui Chen, Martin Fikácek

**Affiliations:** 1 Institute of Entomology, Life Science School, Sun Yat-sen University, Guangzhou, 510275, Guangdong, China; 2 Department of Entomology, National Museum, Cirkusová 1740, Praha 9, CZ - 19100, Czech Republic

**Keywords:** Aquatic beetles, new combination, new synonym, Oriental Region, Palearctic Region, water scavenger beetle.

## Abstract

A new species of the water scavenger beetle, Laccobius (Glyptolaccobius) motuoensis**sp. nov.**, is described from Motuo County, Xizang, China and its diagnostic characters are illustrated. Examination of this new species and re-examination of previously described species revealed that the separation of the subgenus Glyptolaccobius Gentili, 1989 and *Cyclolaccobius* Gentili, 1991 is artificial: both subgenera are hence combined here. *Cyclolaccobius***syn. nov.** is synonymized with *Glyptolaccobius*, and the latter is shown to be diagnosed by 7-segmented antennae as a unique synapomorphy. All species treated until now under *Cyclolaccobius* are here transferred to *Glyptolaccobius*, with the only exception of *L. hingstoni* Orchymont, 1926, *L. jumlanus* Gentili, 2015 and *L. zugmayeri* Knisch, 1910 which are tentatively transferred to the subgenus Hydroxenus Wollaston, 1867, as their antennae bear eight antennomeres. Three species are recorded for the first time from China: L. (Microlaccobius) orientalis Knisch, 1924 from Xizang, Laccobius (M.) exilis Gentili,1974 from Xinjiang, and Laccobius (M.) sublaevis J. Sahlberg, 1900 from Xinjiang. Additional faunistic data from China are provided for the following species: L. (Microlaccobius) hammondi Gentili, 1984, Laccobius (M.) formosus Gentili, 1979, Laccobius (Hydroxenus) hingstoni d’Orchymont, 1926, Laccobius (Glyptolaccobius) yunnanensis Gentili, 2003, Laccobius (Compsolaccobius) decorus (Gyllenhal, 1827), Laccobius (Dimorpholaccobius) bipunctatus (Fabricius, 1775), Laccobius (D.) striatulus (Fabricius, 1775), Laccobius (s. str.) bedeli Sharp, 1884, L. (s. str.) binotatus d’Orchymont, 1934, Laccobius (s. str.) cinereus Motschulsky, 1860, and Laccobius (s. str.) minutus (Linnaeus, 1758).

## Introduction

Since the species of the genus *Laccobius* Erichson, 1837 occurring in China and neighboring areas were reviewed by Gentili (1995, 2003) and Jia et al. (2013a), and only three species of this genus were described from this region more recently (Jia et al. 2013b; Gentili 2015; Zhang and Jia 2017). The fauna of the eastern Himalayas and neighboring mountain systems have not yet been investigated properly and the discovery of additional species is expected.

In total, 34 species of *Laccobius* have been recorded from China to date (Hansen 1999; Short and Fikáček 2011; Fikáček et al. 2015; Zhang and Jia 2017). Here, we summarize new findings based on the material collected in Xinjiang, Qinghai, Xizang and northwestern Sichuan during 2016–2018 deposited in the collection of Sun Yat-sen University, Guangzhou, China. A new species is described and three species are reported from China for the first time. Additional faunistic records are provided for several other species. The total number of *Laccobius* species occurring in China now rises to 38. The examination of the new species also urged us to re-examine the antennal morphology of the species assigned to the subgenera *Glyptolaccobius* Gentili, 1989 and *Cyclolaccobius* Gentili, 1991; this is revealed to be unique for these two subgenera indicating that they should be combined here into a single subgenus.

## Material and methods

A few specimens were dissected for each species examined. After 8–10 hours in 10% KOH at room temperature, male genitalia were transferred to a drop of distilled water, remaining membranes were removed under a compound microscope, and the cleaned genitalia was subsequently mounted into a drop of glycerin on a piece of transparent plastic attached below the respective specimen. Habitus photographs were taken using a Zeiss Discovery V20 with Zeiss AxioCam HRc. Photographs of genitalia were taken using Zeiss Axioskop 40 binocular microscope with a QIMAGING Micropublisher 3.3 RTV camera; the pictures were subsequently combined with the Auto-Montage software. Scanning electron microscope photographs were taken using a Phenom Pro SEM.

Morphological terminology largely follows Gentili and Fikáček (2009) and Jia et al. (2013a).

Examined specimens are deposited in the following collections:

**NMPC**National Museum, Prague, Czech Republic;

**SYSU**Biological Museum, Sun Yat-sen University, Guangzhou, China.

## Results

### Description of new species

#### Laccobius (Cyclolaccobius) motuoensis sp. nov.

Taxon classificationAnimaliaColeopteraHydrophilidae

A92F1DE7-881F-5172-A601-6400C4C47E75

http://zoobank.org/1D637BE4-2A99-4400-8386-9B6B25C87EE3

Figs 1–6 , 7–16 , 17–22 , 23–25

##### Type locality.

China, Xizang, Linzhi Prefecture, Motuo County, Lagong, 29°18'50"N, 95°19'07"E.

##### Type material.

***Holotype***: ♂ (SYSU): China, Xizang, Linzhi Prefecture, Motuo County, Lagong (中国, 西藏, 林芝, 墨脱县, 拉贡), 29°18'50"N, 95°19'07"E, 1271 m. 22.vi. 2018, Shi-shuai Wang & Zu-long Liang let. (transcribed from Chinese). ***Paratypes*** (47 spec.): 39 spec. (SYSU, NMPC): same label data as the holotype; 8 spec. (SYSU): China, Xizang: near road of Motuo to Bomi, Wudang waterfall, 18.viii.2018, Run Zhou.

##### Diagnosis.

Length 1.9–2.2 mm. Dorsal surface dark brown or black with broad lateral yellow band on pronotum and elytra, posterior half of elytra yellowish. Head and pronotum without shagreen on interstices. Head without pale preocular spots. Antenna with seven antennomeres, the third antennomere very small, globular. Elytra often with a pair of yellow spots on the base of third primary series of punctures. Elytra without sulci, with 10 primary series of punctures; primary series of punctures strong and coarse, secondary ones consisting of smaller and more scarcely arranged punctures, punctures with short yellow setae. Aedeagus: total length 0.45 mm; median lobe as long as parameres, broad basally, narrowest medially, subapically with a series of backward directed setae; parameres subrectangular apically, almost as wide as medial lobe at apex.

##### Description.

**Figures 1–6.**
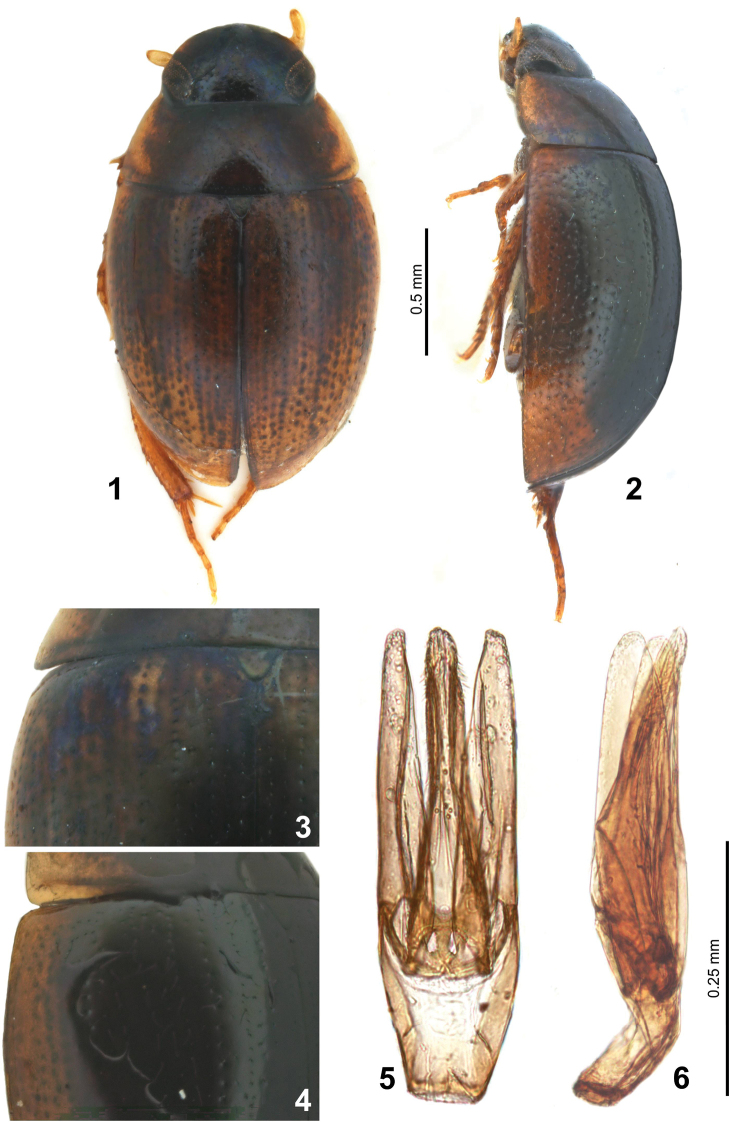
Laccobius (Glyptolaccobius) motuoensis sp. nov. **1, 2** habitus (**1** dorsal view **2** lateral view) **3, 4** base of the elytron (**3** specimen with yellow spot **4** specimen without yellow spot) **5, 6** male genitalia (**5** dorsal view **6** lateral view).

Total length 1.9–2.2 mm (holotype: 2.15 mm); maximum width 1.35–1.45 mm (holotype: 1.4 mm). Total length / total width ratio = 1.5. Body oval, moderately convex, maximum width at anterior third of elytra (Fig. 1).

**Figures 7–16.**
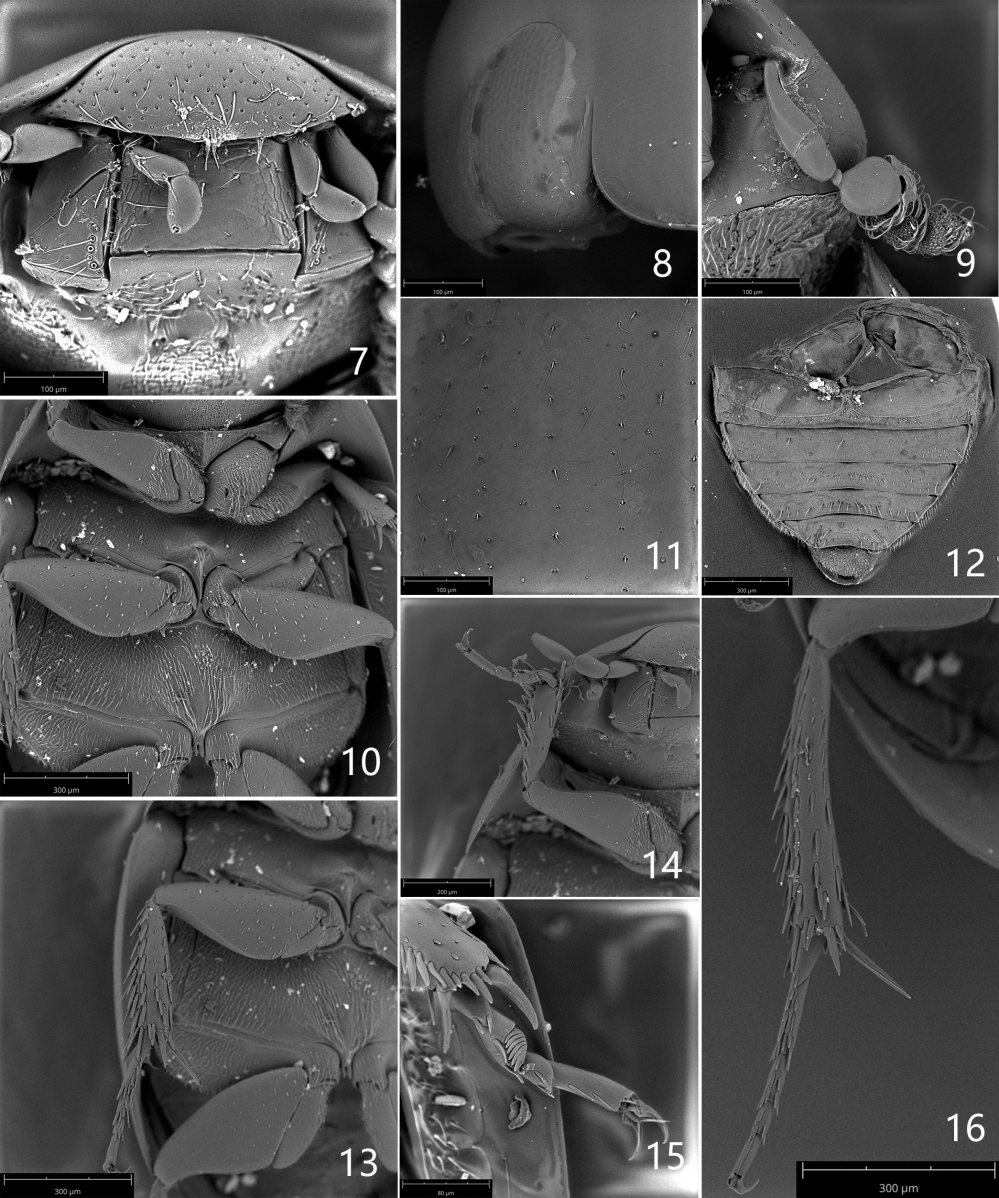
Laccobius (Glyptolaccobius) motuoensis sp. nov. **7** head, ventral view **8** compound eye in lateral view **9** antenna **10** ventral view of thorax **11** punctures on elytral base **12** abdomen **13** mesothoracic leg **14** prothoracic leg **15** male protarsus **16** metathoracic leg.

***Head.*** Black with greenish reflection, without preocular spots, smooth. Labrum about 2.7× as wide as long, without specula in both sexes, feebly emarginated on anterior margin, surface densely punctured (Fig. 7). Clypeus and frons blackish, surface with irregularly arranged punctures, punctures sparse and less impressed than on labrum, each puncture with decumbent white seta on disc, punctures in anterior corner of clypeus as dense as on labrum. Frontoclypeal suture scarcely apparent. Eyes oblong, oblique (Fig. 8), closest to each other posteriorly, slightly protruding laterally, minimum interocular distance in dorsal view 2.7× the width of one eye. Mentum with mesh-like microsculpture anteriorly and laterally (Fig. 7), without punctures, but smooth and with a few punctures in posterior half; submentum smooth with sparse punctures. Maxillary palpi (Fig. 14) yellowish, not becoming dark at extreme apex; palpomere 2 almost same length as palpomere 3, palpomere 4 asymmetrical, inner margin straight and outer margin convex, ca. 1.5× as long as palpomere 3. Apical labial palpomere asymmetrical with straight inner face and convex outer face (Fig. 7), about as long as penultimate one. Antennae with 7 antennomeres (Fig. 9), yellow brown with antennal club of the same color; scape ca. 2.2× as long as pedicel, antennomere 3 very small, globular, cupule globular and asymmetrical; antennomeres 5–7 with densely arranged setae, antennomere 7 constricted near apex.

***Thorax.*** Pronotum transverse, smooth; black with greenish reflection, lateral margins with yellow stripe that extends to posterior margin near posterior corner (Figs 1, 2), the yellow patch with posterior margin 2× as wide as anterior margin; punctures coarse and sparse, bearing decumbent yellowish setae; lateral stria fine, ending in posterior corner, but shortly continuous along anterior margin. Scutellar shield equilaterally triangular, black with few punctures. Prosternum black with dense decumbent pubescence, with a longitudinal keel medially (Fig. 10). Mesoventrite with arrow-head-shaped elevation (Fig. 10), the top of the elevation with a tuft of long setae; a longitudinal carina reaching to posterior margin of mesocoxae (Fig. 10). Metaventrite pubescent with a very narrow longitudinal glabrous area medioposteriorly (Fig. 10). Elytra smooth, slightly elongate, ca. 1.1× as long as wide, dark brown with wide lateral yellow margin that always narrower than posterior margin of pronotal lateral yellow margin (Figs 1, 4); posterior half yellow, fused with lateral yellow margin (Figs 1, 2); base of elytra with a pair of distinct pale yellow spots ca. at mid width (Figs 1, 3), sometimes elytra almost black on disc, and the basal yellow spot absent or unclear (Fig. 4); primary series punctures not sulciform; primary series of punctures strong and coarse, regularly arranged; secondary ones with small and scarce punctures (Fig. 11). Epipleura oblique, ending at level of metafemora.

***Legs.*** Yellow brown. Trochanters pubescent; profemora with anterior surface densely pubescent basally, with tibial grooves; protibiae smooth, bearing stiff setae (Fig. 14). Mesotrochanters and mesofemora smooth (Fig. 13), the latter with tibial grooves; mesotibiae with longitudinal rows of stiff setae (Fig. 13). Metatrochanters and metafemora smooth (Fig. 13), the latter with tibial grooves; metatibiae with longitudinal rows of stiff setae, long spur as long as first and second tarsomeres combined (Fig. 16). Tarsal natatory setae nearly absent. Legs with five tarsomeres; second metatarsomere ca. 1.4× as long as metatarsomere 3 (Fig. 16).

***Abdomen.*** Ventrites 1–5 smooth, without microsculpture, sparsely pubescent; each ventrite with long setae posteriorly, ventrite 6 densely pubescent (Fig. 12).

***Male.*** Second protarsomere dilated, with a clasping structure that contains 7 lobes (Fig. 15).

***Aedeagus.*** (Figs 5, 6). Total length 0.42 mm. parameres nearly 1.6× as long as phallobase. Phallobase 1.3× as long as wide. Median lobe as long as parameres, broad basally, gradually narrowed from base to mid length and then slightly widened to apex; with a series of backward directed setae subapically, apex rounded. Parameres subrectangular apically, almost as wide as medial lobe apically.

##### Differential diagnosis.

This species closely resembles *L. yunnanensis* Gentili, 2003 and *L. sipeki* Gentili & Fikáček, 2009 in the genital morphology (including the series of long hairs on distal half of the median lobe) and the coloration of elytra having a dark base with small basal spots and widely pale apical portion. It can be distinguished from *L. yunnanensis* by having an elytral series regular throughout (somewhat irregular at base in *L. yunnanensis*), primary series of punctures distinctly stronger and coarser than secondary ones (with some punctures at least as large as those on primary series in *L. yunnanensis*) and the median lobe ca. the same width in apical half (distinctly widened apically in *L. yunnanensis*). It can be distinguished from *L. sipeki* by the absence of the parasutural furrow (with distinct rather deep parasutural groove in *L. sipeki*) and the apex of the median lobe reaching the level of parameral apices only (slightly overlapping parameral apices in *L. sipeki*).

##### Etymology.

This species is named after type locality.

##### Distribution.

Only known from two close localities in the eastern Himalaya (Xizang, Motuo County).

### Synonymy of the subgenera *Glyptolaccobius* and *Cyclolaccobius*

The subgenera *Glyptolaccobius* and *Cyclolaccobius* both contain species that inhabit mostly hygropetric habitats (seepages, wet rocks, sides of waterfalls) and are both characterized by large transverse eyes, which distinguish them from remaining groups of the genus *Laccobius*. The principal character distinguishing both subgenera is the presence (in *Glyptolaccobius*) or absence (in *Cyclolaccobius*) of the ‘parasutural furrow’, i.e., the longitudinal impression situated parallel to the suture in the posterior half of elytra. This character is usually clearly visible in *Glyptolaccobius* and clearly absent in *Cyclolaccobius*, but confusion still happens: Gentili (2006) and Gentili and Fikáček (2009) described three species of *Glyptolaccobius* (*L. silvester* Gentili, 2006, *L. hanka* Gentili & Fikáček, 2009 and *L. josefi* Gentili & Fikáček, 2009) which were later transferred to *Cyclolaccobius* by Gentili (2012). Both subgenera were mentioned as bearing 8-segmented antenna, similarly to all other *Laccobius* species (see Gentili 2006, fig. 3).

Recently, Zhang and Jia (2017) described a new *Glyptolaccobius* species from Yunnan, China (*L. yinziweii* Zhang & Jia, 2017) which is unusual among all known *Laccobius* as its antenna consisted of seven antennomeres only: scape, pedicel, one intermediate antennomere (instead of two in other *Laccobius* species), cupule and three-segmented antennal club (see Zhang and Jia 2017: figs 8, 9) showing scanning electron micrographs of the antennae). The examination of the antennal morphology of *L. motuoensis* described in this paper surprisingly revealed the same morphology with only seven antennomeres. In contrast to *L. yinziweii*, *L. motuoensis* clearly lacks the parasutural furrow, and both species sharing the unique antennal morphology are hence members of different subgenera. Moreover, the genital morphology of *L. motuoensis* closely resembles that of *L. yunnanensis* (a member of *Cyclolaccobius*) and *L. sipeki* (member of *Glyptolaccobius*). In addition, the elytral coloration of *L. motuoensis*, with pale spots basally, completely dark portion subbasally and at mid length and a pale portion in the apical third to fourth of the elytra, is found in a group of species of which some are treated as *Glyptolaccobius* (*L. sipeki*, *L. yinziweii*) and some as *Cyclolaccobius* (*L. josefi*, *L. hanka* and *L. nigrogilvus*).

All these observations motivated us to check the antennal morphology of a wider spectrum of species assigned to *Glyptolaccobius* and *Cyclolaccobius*. We examined the following species: *L. affinis* Knisch, 1927 (the type species of *Glyptolaccobius*), *L. pluvialis* Gentili, 2006, *L. qinlingensis* Jia, Gentili & Fikáček, 2013, *L. sipeki* Gentili & Fikáček, 2009, *L. yinziweii* Zhang & Jia, 2017 (all latter with the parasutural furrow, i.e., members of *Glyptolaccobius*), *L. hanka* Gentili & Fikáček, 2009, *L. hainanensis* Jia, Gentili & Fikáček, 2013, *L. hingstoni* Orchymont, 1926, *L. martini* Jia, Song & Gentili, 2013, *L. nitidus* Gentili, 1984, *L. politus* Gentili, 1979, and *L. yunnanensis* Gentili, 2003 (all latter without parasutural furrow, i.e., members of *Cyclolaccobius*). All of these except *L. hingstoni* have 7-segmented antennae. Moreover, *L. hanka* (without parasutural furrow, member of *Cyclolaccobius*) and *L. pluvialis* (with parasutural furrow, member of *Glyptolaccobius*) were found as closely related sister taxa in the molecular phylogeny of Toussaint and Short (2018). All this evidence clearly indicates that species assigned at the moment to the subgenera *Glyptolaccobius* and *Cyclolaccobius* likely form a monophyletic group characterized by a unique synapomorphy within *Laccobius*, i.e., the 7-segmented antenna. The presence/absence of the parasutural furrow seems to be a phylogenetically flexible character within *Laccobius* since species that closely resemble each other in other characters sometimes differ in the presence/absence of the parasutural furrow only. We hence conclude that keeping *Glyptolaccobius* and *Cyclolaccobius* as two subgenera is actually more confusing than helpful for taxonomic work, even if these subgenera would be considered just as artificial groups designed to facilitate taxonomic work on this large genus (until a phylogenetic study is performed). For this reason, we are performing the following taxonomic changes here:

1. We synonymize *Glyptolaccobius* Gentili, 1989 with *Cyclolaccobius* Gentili, 1991 syn. nov.

2. *Glyptolaccobius* sensu nov. is diagnosed by having antennae with seven antennomeres (in contrast to eight antennomeres in remaining subgenera of *Laccobius*). All species currently treated under *Cyclolaccobius* and having 7-segmented antennae are here transferred to *Glyptolaccobius*.

3. The only three species treated until now as *Cyclolaccobius* which do not have 7-segmented antennae (i.e., *L. hingstoni*, *L. zugmayeri* Knisch, 1910 and *L. yumlanus* Gentili, 2015; see Gentili 2015) are tentatively transferred to the subgenus Hydroxenus Wollaston, 1867 (= *Platylaccobius* Gentili, 1974) where they were assigned originally before being transferred to *Cyclolaccobius*. These three species differ from other *Cyclolaccobius* and *Glyptolaccobius* species by having a much larger body size, elongate oval body, eyes not so transversely reniform and by their biology: *L. hingstoni* was collected in the littoral zone of standing waters (ponds, lakes) i.e. not in hygropetric habitats or habitats associated with stony streams which are typical for the *Glyptolaccobius*+*Cyclolaccobius* species.

Additional studies are necessary to understand the systematics of *Laccobius* and to test the phylogenetic position of *L. zugmayeri* and related species as well as to test the monophyly of the subgenera. At the moment, only the subgenus Glyptolaccobius in the new meaning (i.e., containing all *Laccobius* species with 7-segmented antenna, and characterized by a hygropetric lifestyle) and *Yateberosus* (a New Caledonia endemic subgenus with the larva having closed spiracular system and abdomen bearing tracheal gills, see Fikáček et al. 2018) seem to be supported by unique morphological synapomorphies combined with geographically limited range (in *Yateberosus*) or a specific lifestyle (in *Glyptolaccobius*), and hence are candidates for monophyletic groups. The monophyly of all other subgenera is doubtful and needs to be further tested.

### Key to species of Laccobius (Glyptolaccobius) from China

Gentili (1995) provided a series of keys for the identification of all *Laccobius* species known from China and neighboring areas. These keys are still up-to-date for most subgenera and include even the species recorded subsequently as new for China (Jia et al. 2013; this paper, see below). Only two parts need to be updated: (1) *Laccobius jumlanus* Gentili, 2015 was mistakenly treated under the name *L. zugmayeri* Knisch, 1910 in the key (see Gentili 2015 for details); and (2) the key of species of the subgenus Glyptolaccobius sensu nov. needs to be updated as all species described as new since 1995 belong to this subgenus. The following key to *Glyptolaccobius* sensu nov. in China is modified from Gentili (1996, 1998).

**Figures 17–22.**
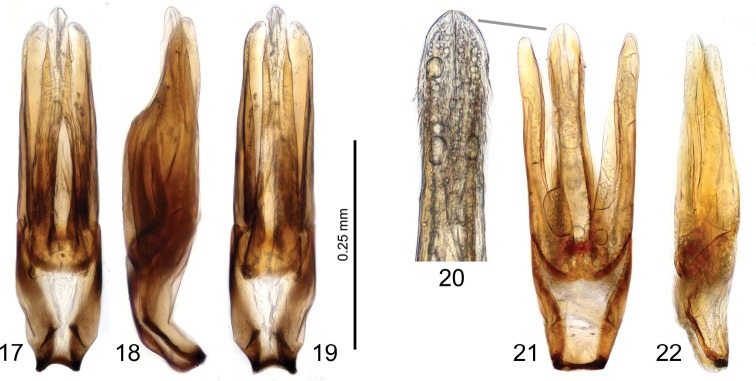
Male genitalia of the species similar to *Laccobius motuoensis* sp. nov. **17–19***L. sipeki* Gentili & Fikáček, holotype (**17** ventral **18** lateral **19** ventral) **20–22***L. yunnanensis* Gentili, specimen from China: Yunnan: Rehai Hot springs (coll. NMPC) **20** detail of the apical half of medial lobe **21** ventral view **22** lateral view.

**Table:** 

1	Elytra with distinct parasutural furrows	**2**
–	Elytra without distinct parasutural furrows	**3**
2	Body length 2.7–2.9 mm; elytra without pale yellow spots at base; median lobe bearing lateral subapical rows of stout spines (Jia et al, 2013: figs 3–5)	***L. qinlingensis* Jia, Gentili & Fikáček**
–	Body length 1.8–2.1 mm; base of elytra with two or four distinct pale yellow spots; median lobe without lateral subapical rows of spines (Zhang and Jia 2017: figs 6, 7)	***L. yinziweii* Zhang & Jia**
3	Body length 2.0–2.8 mm; elytral borders and epipleura are swollen at base; median lobe narrowly pointed apically (Gentili 1995: figs 52, 53)	***L. nitidus* Gentili**
–	Body length 1.8–2.1 mm; elytral borders and epipleura are not swollen at base; median lobe rounded apically	**4**
4	Apical part of the median lobe without series of fine setae. Elytra completely black at the base. Species from Hainan and Taiwan	**5**
–	Apical part of the median lobe with series of fine setae. Elytra with or without pale basal spots. Continental species	**6**
5	Median lobe strongly constricted subapically (Jia et al. 2013: figs10–12). Hainan	***L. hainanensis* Jia, Gentili & Fikáček**
–	Median lobe indistinctly constricted subapically (Jia et al. 2013: figs 14–16). Taiwan	***L. politus* Gentili**
6	Elytral series regular throughout; primary series of punctures distinctly stronger and coarser than secondary ones; median lobe ca. of the same width in apical half (Figs 5, 6)	***L. motuoensis* sp. nov**.
–	Elytral series somewhat irregular at base, with some secondary punctures at least as large as those on primary series; median lobe distinctly widened apically (Figs 20–22)	***L. yunnanensis* Gentili**

**Figures 23–25.**
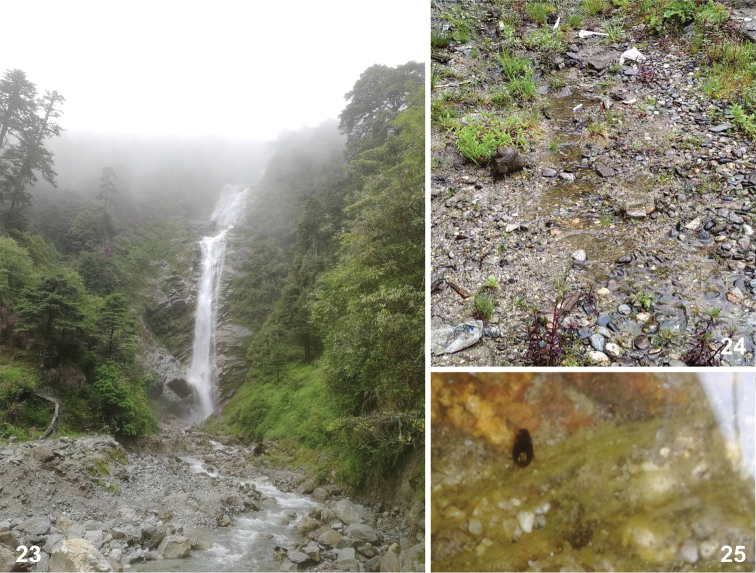
Habitats of *Laccobius motuoensis* sp. nov. Xizang: near road of Motuo to Bomi, Wudang waterfall.

### Additional faunistic data to China

#### Laccobius (Microlaccobius) orientalis

Taxon classificationAnimaliaColeopteraHydrophilidae

Knisch, 1924

B4774FC0-A4CE-5DFA-B688-519F55289729

Fig. 27

##### Material examined.

**XIZANG**: 41 spec. (SYSU): Linzhi, Bomi County, Hagu township, Taohuagou, 29°59'42"N, 95°37'15"E, 2673 m, 20.vi.2018, Shi-shuai Wang & Zu-long Liang leg.

##### Diagnosis.

Body length 2.8–3.0 mm. Frons between eyes ca. 2.6–3.0× as wide as one eye in dorsal view. Pronotum black or dark brown medially, with a pair of light spots at anterior margin, with broad yellow band laterally, without shagreen, smooth and shining. Punctures of third and fifth elytral rows uniform, arranged almost in a straight line (a few punctures slightly out of line). Aedeagus (Fig. 27): Basal two-thirds of parameres almost parallel-sided, gradually narrowed towards apex in apical one-third, pointed apically; median lobe almost as wide as parameres basally, gradually narrowed towards apex, rounded apically.

##### Distribution.

Widely distributed species, recorded from north Africa through Near East and Central Asia to the Himalaya and Tibetan Plateau (Hansen 1999, Fikáček et al. 2015). **New for China.**

#### Laccobius (Microlaccobius) exilis

Taxon classificationAnimaliaColeopteraHydrophilidae

Gentili, 1974

8015776A-4965-5A90-BE77-40F3F276CF4A

Fig. 26

##### Material examined.

**XINJIANG**: 15 spec. (SYSU): Yili, Chabuchar, beside road, 43.77N, 81.15E, 574 m, 4.vii.2017, Rui-juan Zhang & Shi-shuai Wang leg.; 1 male (SYSU): Altai, Habahe County, Baishahu, 48.37N, 85.74E, 553 m, 10.vii.2017, Rui-juan Zhang & Shi-shuai Wang leg.

##### Diagnosis.

**Figures 26–28.**
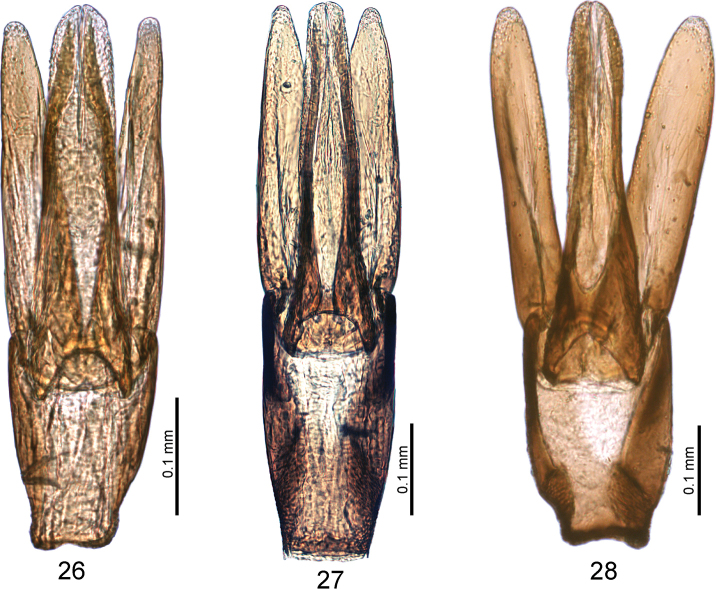
Aedeagi of the species newly recorded from China (all based on Chinese specimens): **26***Laccobius exilis* Gentili **27***L. orientalis* Knisch **28***L. sublaevis* Sahlberg.

Body length 2.3–2.7 mm. Frons between eyes ca. 2.8–3.0× as wide as one eye in dorsal view. Pronotum black or dark brown medially, without a pair of light spots at anterior margin, with broad yellow band laterally, without shagreen, smooth and shining. Punctures of third and fifth elytral rows uniform, arranged almost in a straight line (Gentili 1995: fig. 162). Aedeagus (Fig. 26) with parameres narrowed towards apex, rounded apically; median lobe wide throughout, slightly wider than parameres, especially in apical part.

##### Distribution.

Widely distributed species, recorded from Turkey through Central Asia to the Himalaya, Myanmar and the Tibetan Plateau (Gentili 1995, Hansen 1999, Fikáček et al. 2015). **New for China.**

#### Laccobius (Microlaccobius) sublaevis

Taxon classificationAnimaliaColeopteraHydrophilidae

J. Sahlberg, 1900

DD552B94-0A8F-59A1-A03A-C907AD94B657

Fig. 28

##### Material examined.

**XINJIANG**: 1 male (SYSU): Bayinguoleng Mongolian Autonomous Prefecture, Hejing County, Gongnais Forest Farm, 43°14'15"N, 84°39'41"E, 2019 m, 1.vii.2017, Rui-juan Zhang, Shi-shuai Wang, Kai Chen & Yong-jiang Duan leg. 1 male (SYSU): Gongnais valley, 31.vii.2005, Ling Zhao leg.

##### Diagnosis.

Size 2.8–3.0 mm. Frons between eyes ca. 2.8–3.0× as wide as one eye. Pronotum black or dark brown medially, without a pair of light spots at anterior margin, with broad yellow band laterally, without shagreen, smooth and shining. Punctures of third and fifth elytral rows in disorder, not arranged in straight lines (Gentili 1995: fig. 161). Aedeagus (Fig. 28) with median lobe slightly widened apically; parameres with subparallel sides, rounded apically.

##### Distribution.

Central Asian species reaching to the Tibetan Plateau and the Himalaya Region (Gentili 1995, Fikáček et al. 2015). **New for China.**

#### Laccobius (Microlaccobius) hammondi

Taxon classificationAnimaliaColeopteraHydrophilidae

Gentili, 1984

63F34D5D-7E89-5CE5-8810-2D050BBC3510

##### New material examined.

**SICHUAN**: 3 spec. (SYSU): Luding County, Moxi town, Yuejin village, a pool with sands, 29°44'6.1"N, 102°04'5.6"E, 2150 m, 30.vi–1.vii.2016, F.-L.Jia, R.B. Angus, Kai Chen, Z.-Q. Li leg.

##### Distribution.

See Fikáček et al. (2015). **New for Qinghai-Tibetan Plateau.**

#### Laccobius (Microlaccobius) formosus

Taxon classificationAnimaliaColeopteraHydrophilidae

Gentili, 1979

0F75F204-6943-55FA-818A-180C7244E35D

##### New material examined.

**SICHUAN**: 3 spec. (SYSU): Dayi County, Shitouhe River, 30°34–38'N, 102°16–23'E, 616–766 m, 4.vii.2017, F.-L. Jia & R.B. Angus leg.

##### Distribution.

See Fikáček et al. (2015). **New for Sichuan.**

#### Laccobius (Hydroxenus) hingstoni

Taxon classificationAnimaliaColeopteraHydrophilidae

d’Orchymont, 1926

2DAFC103-B486-5400-96A2-200858BF1F31

##### New material examined.

**XIZANG**: 11 spec. (SYSU): Rikaze Prefecture, Jiangzi County, Zijin Wetland Park, 28°56'21"N, 89°33'50"E, 3974 m, 1.vii.2018, Shi-shuai Wang & Zu-long Liang leg.; 16 spec. (SYSU, NMPC): Shannan Prefecture, Langkazi County, Haweng village, 28°58'54"N, 90°23'44"E, 4432 m, 2.vii.2018, Shi-shuai Wang & Zu-long Liang leg.

##### Distribution.

See Fikáček et al. (2015).

#### Laccobius (Cyclolaccobius) yunnanensis

Taxon classificationAnimaliaColeopteraHydrophilidae

Gentili, 2003

CB4470AC-61E0-5D99-829C-0933DAB51A96

##### New material examined.

**YUNNAN**: 5 spec. (SYSU): Lushui County, Pianma town, 26.01N, 98.62E, 1908 m, 19.v.2016. Yu-dan Tang & Rui-juan Zhang leg.

##### Distribution.

China (Yunnan: Lushui, Mojiang), Myanmar.

#### Laccobius (Compsolaccobius) decorus

Taxon classificationAnimaliaColeopteraHydrophilidae

(Gyllenhal, 1827)

121F30A0-BC1E-5A80-85FE-B78F57AB15BD

##### New material examined.

**QINGHAI**: 1 male (SYSU), near Qinghaihu Lake, 27.vii.2017, Yang Liu leg.

##### Distribution.

See Fikáček et al. (2015).

#### Laccobius (Dimorpholaccobius) bipunctatus

Taxon classificationAnimaliaColeopteraHydrophilidae

(Fabricius, 1775)

356F0894-7C0C-50D1-9B5E-F9C16EC89279

##### New material examined.

**XINJIANG**: 9 spec. (SYSU): Altai prefectures, Xiaodonggou Forest Park, 47.94N, 88.15E, 1108 m, 12.vii.2017, Rui-juan Zhang & Shi-shuai Wang leg. 22 spec. (SYSU), Bayinguoleng Mongolian Autonomous Prefecture, Hejing County, Gongnais Forest Farm, 43°14'15"N, 84°39'41"E, 2019 m, 1.vii.2017, Rui-juan Zhang, Shi-shuai Wang, Kai Chen & Yong-jiang Duan leg. 12 spec. (SYSU), Altai, Bahahe County, Baihualin, 48.07N, 86.34E, 512 m, 7.vii.2017, Rui-juan Zhang & Shi-shuai Wang leg. 2 exs. (SYSU), Altai, Habahe County, Baishahu, 48.37N, 85.74E, 553 m, 10.vii.2017, Rui-juan Zhang & Shi-shuai Wang leg. 8 exs. (SYSU), Tacheng Prefecture, Natural Forest, 46.37N, 85.74E, 1042 m, 10.vii.2017, Rui-juan Zhang & Shi-shuai Wang leg. 15 exs. (SYSU), Yili Kazak Autonomous Prefecture, Xinyuan County, Natural forest, 43°22'54"N, 83°33'52"E, 1282m, 2.vii.2017, Rui-juan Zhang, Shi-shuai Wang, Kai Chen & Yong-jiang Duan leg. 2 exs. (SYSU), Altai Prefecture, Burjin County, Kanas, 48.31N, 87.11E, 1336 m, 11.vii.2017, Rui-juan Zhang & Shi-shuai Wang leg. 4 exs. (SYSU), Tacheng Prefecture, Yikekure village, 46.81N, 85.98E, Rui-juan Zhang & Shi-shuai Wang leg.

##### Distribution.

See Fikáček et al. (2015).

#### Laccobius (Dimorpholaccobius) striatulus

Taxon classificationAnimaliaColeopteraHydrophilidae

(Fabricius, 1775)

22A4A2C3-BD00-59A6-BEC9-C6D7BD2ECFFD

##### New material examined.

**XINJIANG**: 6 exs. (SYSU), Tacheng Prefecture, Emin County, Shuimogou, 46.39N, 83.93E, 863 m, 6.vii.2017, Rui-juan Zhang & Shi-shuai Wang leg. 40 spec. (SYSU), Altai Prefecture, Keketuohai wetland, 47°01'16"N, 89°45'22"E, 1343 m, 13.vii.2017, Rui-juan Zhang, Shi-shuai Wang, Kai Chen & Yong-jiang Duan leg. 108 spec. (SYSU), Altai, Habahe County, Baishahu, 48.37N, 85.74E, 553 m, 10.vii.2017, Rui-juan Zhang & Shi-shuai Wang leg. 16 spec. (SYSU), Altai, Bahahe County, Baihualin, 48.07N, 86.34E, 512 m, 7.vii.2017, Rui-juan Zhang & Shi-shuai Wang leg. 5 spec. (SYSU), Habahe County, Tiereketi Town, 48.42N, 86.73E, 1242 m, 10.vii.2017, Rui-juan Zhang & Shi-shuai Wang leg. 3 spec. (SYSU), Altai Prefecture, a small pool beside road, 47.86N, 88.10E, 1107 m, Rui-juan Zhang & Shi-shuai Wang leg. 4 spec. (SYSU), (SYSU), Bayinguoleng Mongolian Autonomous Prefecture, Hejing County, Gongnais Forest Farm, 43°14'15"N, 84°39'41"E, 2019 m, 1.vii.2017, Rui-juan Zhang, Shi-shuai Wang, Kai Chen & Yong-jiang Duan leg. 2 spec. spec. (SYSU), Tacheng Prefecture, Yikekure village, 46.81N, 85.98E, Rui-juan Zhang & Shi-shuai Wang leg.

##### Distribution.

See Fikáček et al. (2015).

#### Laccobius (s. str.) bedeli

Taxon classificationAnimaliaColeopteraHydrophilidae

Sharp, 1884

FC846094-D9FB-5D37-84F7-8CF44C4D20E0

##### New material examined.

**XINJIANG**: 1 male (SYSU), Altai, Habahe County, Baishahu, 48.37N, 85.74E, 553 m, 10.vii.2017, Rui-juan Zhang & Shi-shuai Wang leg.

##### Distribution.

See Fikáček et al. (2015). **New for Xinjiang.**

#### Laccobius (s. str.) binotatus

Taxon classificationAnimaliaColeopteraHydrophilidae

d’Orchymont, 1934

8603D24D-F171-5986-825D-CF1A5B566107

##### New material examined.

**QINGHAI**: 12 spec. (SYSU), Huangzhong County, Kangoumen village, 36°29'24"N, 101°40'2"E, 2502 m, 28.viii.2018, Zu-long Liang & Jun-wei Deng leg.; 3 spec. (SYSU), Huangzhong County, Yajia village, 36°27'41"N, 101°42'38"E, 2610 m, 28.viii.2018, Zu-long Liang & Jun-wei Deng leg.; 4 spec. (SYSU), Huangzhong County, Shangshanzhuang Village, 36°26'33"N, 101°40'51"E, 2674 m, 28.viii.2018, Zu-long Liang & Jun-wei Deng leg.; 4 spec. (SYSU), Ping’an County, Baijiacun village, 36°26'46"N, 102°3'28"E, 2240 m, 29.viii.2018, Zu-long Liang & Jun-wei Deng leg.; 1 male (SYSU), Huzhu County, Nanmenxian town, 36°59'21"N, 101°54'9"E, 2910 m, 30.viii.2018, Zu-long Liang & Jun-wei Deng leg. **SICHUAN**: 9 spec. (SYSU), Luding County, Moxi town, Yuejin village, a pool with sands, 29°44'6.1"N, 102°04'5.6"E, 2150 m, 30.vi-1.vii.2016, Fenglong Jia, Robert, R.B. Angus, Kai Chen & Zhi-qiang Li leg.

##### Distribution.

See Jia et al. (2013a) and Fikáček et al. (2015). **New for Sichuan.**

#### Laccobius (s.str.) cinereus

Taxon classificationAnimaliaColeopteraHydrophilidae

Motschulsky, 1860

7BB71176-F508-59D3-A631-BA9779E83561

##### New material examined.

**QINGHAI**: 19 spec. (SYSU), Maduo, Yematan, 34°42'5"N, 98°4'5"E, 3178 m, 21.viii.2018, Zu-long Liang & Jun-wei Deng leg.; 15 spec. (SYSU), Maduo, Donggecuona Lake, 35°17'12"N, 98°42'22"E, 4085 m, 24.vii.2018, Zu-long Liang & Jun-wei Deng leg.; 54 spec. (SYSU), Gangcha, beside national road G135, 37°17'23"N, 100°14'49"E, 3178 m, 21.viii.2018, Zu-long Liang & Jun-wei Deng leg. **XINGJIANG**: 1 male (SYSU), Altai, Bahahe County, Baihualin, 48.07N, 86.34E, 512 m, 7.vii.2017, Rui-juan Zhang & Shi-shuai Wang leg.

##### Distribution.

See Fikáček et al. (2015). **New for Xinjiang.**

#### Laccobius (s. str.) minutus

Taxon classificationAnimaliaColeopteraHydrophilidae

(Linnaeus, 1758)

FB9F3C05-D0BA-5672-AF2E-78479432C746

##### New material examined.

**XINJIANG**: 23 spec. (SYSU), Altai prefectures, Xiaodonggou Forest Park, 47.94N, 88.15E, 1108 m, 12.vii.2017, Rui-juan Zhang & Shi-shuai Wang leg. 53 spec., (SYSU), Altai Prefecture, Burjin County, Kanas, 48.31N, 87.11E, 1336 m, 11.vii.2017, Rui-juan Zhang & Shi-shuai Wang leg. 92 spec. (SYSU), Altai Prefecture, Habahe County, Baihualin, 48.07N, 86.34E, 512 m, 7.vii.2017, Rui-juan Zhang & Shi-shuai Wang leg. 1 ex. (SYSU), Huocheng County, Daxigou, Fushoushan mount, 44.40N, 80.73E, 1201 m, 4.vii.2017, Rui-juan Zhang & Shi-shuai Wang leg.

##### Distribution.

See Fikáček et al. (2015).

## Supplementary Material

XML Treatment for Laccobius (Cyclolaccobius) motuoensis

XML Treatment for Laccobius (Microlaccobius) orientalis

XML Treatment for Laccobius (Microlaccobius) exilis

XML Treatment for Laccobius (Microlaccobius) sublaevis

XML Treatment for Laccobius (Microlaccobius) hammondi

XML Treatment for Laccobius (Microlaccobius) formosus

XML Treatment for Laccobius (Hydroxenus) hingstoni

XML Treatment for Laccobius (Cyclolaccobius) yunnanensis

XML Treatment for Laccobius (Compsolaccobius) decorus

XML Treatment for Laccobius (Dimorpholaccobius) bipunctatus

XML Treatment for Laccobius (Dimorpholaccobius) striatulus

XML Treatment for Laccobius (s. str.) bedeli

XML Treatment for Laccobius (s. str.) binotatus

XML Treatment for Laccobius (s.str.) cinereus

XML Treatment for Laccobius (s. str.) minutus
